# A High Burden of Asymptomatic Gastrointestinal Infections in Traditional Communities in Papua New Guinea

**DOI:** 10.4269/ajtmh.17-0282

**Published:** 2017-09-25

**Authors:** Paul F. Horwood, Kevin W. Soli, Tobias Maure, Yuichi I. Naito, Ayako Morita, Kazumi Natsuhara, Kiyoshi Tadokoro, Jun Baba, Shingo Odani, Eriko Tomitsuka, Katsura Igai, Jo-Ann Larkins, Peter M. Siba, William Pomat, Emma S. McBryde, Masahiro Umezaki, Andrew R. Greenhill

**Affiliations:** 1 Papua New Guinea Institute of Medical Research, Goroka, Papua New Guinea;; 2 Australian Institute of Tropical Health and Medicine, James Cook University, Cairns, Australia;; 3 Department of Biogeochemistry, Japan Agency for Marine-Earth Science and Technology, Kanagawa, Japan;; 4 Department of Human Ecology, The University of Tokyo, Tokyo, Japan;; 5 Faculty of Nursing, The Japanese Red Cross Akita College of Nursing, Akita, Japan;; 6 Research Institute for Languages and Cultures of Asia and Africa, Tokyo, Japan;; 7 Faculty of Letter, Chiba University, Chiba, Japan;; 8 Department of Health Chemistry, Faculty of Pharmaceutical Sciences, Niigata University of Pharmacy and Applied Sciences, Niigata City, Japan;; 9 Graduate School of Biomedical Sciences, Nagasaki University, Nagasaki, Japan;; 10 School of Applied and Biomedical Sciences, Federation University Australia, Victoria, Australia;; 11 Australian Institute of Tropical Health and Medicine, James Cook University, Townsville, Australia

## Abstract

Stool samples were collected from 148 healthy adults living a traditional subsistence lifestyle in Papua New Guinea and screened for enteric pathogens using real-time RT-PCR/PCR assays. Enteric pathogens were detected in a high proportion (41%) of individuals. Clear differences were observed in the detection of pathogens between highland and lowland communities. In particular, there was a marked difference in detection rates of norovirus GII (20% and 0%, respectively) and *Shigella* sp. (15% and 0%, respectively). Analysis of the relationship between enteric pathogen carriage and microbial community composition of participants, using box plots to compare specific normal flora population numbers, did not suggest that gut microbial composition was directly associated with pathogen carriage. This study suggests that enteric pathogens are common in healthy individuals in Papua New Guinean highland communities, presumably acting as a reservoir of infection and thus contributing to a high burden of gastrointestinal illnesses.

Gastrointestinal infections are a leading cause of morbidity and mortality throughout the world, particularly in children.^1^ In developing countries, where the greatest burden of diarrheal mortalities occur, gastrointestinal infections can also contribute to poor educational outcomes and reduced growth rates in children.^2^ In communities with poor access to sanitation and safe water sources, a cycle of individual infection, followed by pathogen shedding into the environment through diarrhea or asymptomatic excretion, potentially leads to dissemination of infection in the community. This cycle can have major implications on the health and wealth of the community, contributing to the maintenance of the “poverty trap.”^3^ In this study, we investigated the asymptomatic carriage of viral and bacterial enteric pathogens by adults in three traditional populations in remote regions of Papua New Guinea, and searched for correlations between pathogen carriage and previously determined gut microbial community composition data.

Stool samples were collected from participants in two highland regions of Papua New Guinea from February through March 2012 and one lowland site in September 2012, as outlined previously.^4^ Demographic data, history of recent antibiotic use, and dietary information were also collected as previously discussed.^4,5^ Participants were excluded from the study if they reported diarrhea or antibiotic use in the past 2 weeks before sample collection.

Nucleic acids were extracted from stool samples using a modified phenol/chlorophorm extraction method^6^ and tested for a range of bacterial and viral enteric pathogens using previously published real-time PCR/RT-PCR assays (as outlined in Soli et al.^7^). The pathogens targeted in this study were the viruses: rotavirus, norovirus GI and GII, adenovirus types 40 and 41, sapovirus, and astrovirus; and the bacteria: *Salmonella* spp., *Shigella* spp. (or potentially enteroinvasive *Escherichia coli*; EIEC), *Vibrio cholerae*, *Campylobacter* spp., enteropathogenic *E. coli* (EPEC), and enterotoxigenic *E. coli* (ETEC). In addition, eluates from stool samples were also tested for the presence of *Helicobacter pylori* using real-time PCR.^8^ Statistical analyses were calculated in Excel (Microsoft Corporation, Redmond, WA) and SPSS Statistics 20 (IBM Corporation, Armonk, NY).

Fisher’s exact tests were used to determine if associations existed between certain sociodemographic parameters and pathogen carriage. Our group previously used a qPCR approach to investigate the gut microbiota of these human populations.^4^ Pathogen carriage data from this study and the microbiota composition data from our previous study^4^ were analyzed to determine if associations existed between the abundance of certain groups of bacteria and the carriage of enteric pathogens, using box plots to compare specific normal flora population numbers in carriage-positive and carriage-negative participants. These analyses were conducted on all participants for which both pathogen carriage and microbiota results were available (*N* = 115; i.e., all participants who we were able to conduct microbial composition analyses on^4^).

Ethical approval for this study was granted by the PNG Institute of Medical Research Institutional Review Board (Ethics #10.25) and the PNG Medical Research Advisory Council (Ethics #11.25). Stool samples were collected from healthy people after the collection of written informed consent from all participants.

A total of 148 stool samples were collected from healthy adults living a traditional, subsistence lifestyle in rural villages of Papua New Guinea. Samples were collected from two highland regions, the Asaro Valley, Eastern Highlands Province (*N* = 50), and the Tari Basin, Hela Province (*N* = 59); and one lowland region, East Maprik, East Sepik Province (*N* = 39). The demographic and nutritional characteristics of these populations have been described elsewhere.^4,5^

Overall, the most commonly detected pathogens were norovirus GII (15%, *N* = 22), *Shigella* spp. (11%, *N* = 16), EPEC (11%, *N* = 16), *H. pylori* (8%, *N* = 12), ETEC (7%, *N* = 10), norovirus GI (4%, *N* = 6), *Campylobacter* spp. (4%, *N* = 6), and sapovirus (1%, *N* = 2). *Salmonella* spp., *V. cholerae*, adenovirus 40/41, rotavirus, and astrovirus were not detected in any samples. In total, enteric pathogens were detected in 41% (*N* = 61) of the participants.

Significant differences were noted between highland communities versus lowland communities in the carriage rate of any pathogen (*P* value < 0.0001), viral pathogens (*P* value < 0.0001), and bacterial pathogens (*P* value = 0.0027) (Table 1). In particular, the carriage of norovirus GII (20% versus 0%) and *Shigella* sp. (15% versus 0%) was markedly higher in highland versus lowland communities, respectively. Indeed, almost all enteric pathogens were detected only in the two highland communities and not in the lowland communities, with the exception of EPEC and ETEC. There was no observable difference in population numbers of key groups of the normal gut flora in study participants who were pathogen carriage positive compared with those who were negative for the carriage of pathogens (Figure 1).

**Table 1:** The frequency of enteric pathogen detection in healthy participants from traditional communities in Papua New Guinea

	Highland sites			Lowland site‡ (*N* = 39) (%)	Total (*N* = 148) (%)
Characteristics	1* (*N* = 50) (%)	2† (*N* = 59) (%)	Total (*N* = 109) (%)		
Tested positive for a pathogen	54	49	51	13	41
Tested positive for a virus	26	27	27	0	20
Tested positive for a bacteria	40	37	39	13	31
Adenovirus 40/41	0	0	0	0	0
Astrovirus	0	0	0	0	0
Norovirus GI	0	10	6	0	4
Norovirus GII	26	15	20	0	15
Rotavirus	0	0	0	0	0
Sapovirus	0	3	2	0	1
*Campylobacter* sp.	10	2	6	0	4
EPEC§	16	7	11	10	11
ETEC§	0	15	8	3	7
*Helicobacter pylori*	16	7	11	0	8
*Salmonella* sp.	0	0	0	0	0
*Shigella* sp.	12	17	15	0	11
*Vibrio cholerae*	0	0	0	0	0

* Asaro Valley, Eastern Highlands Province.

† Tari Basin, Hela Province.

‡ East Maprik, East Sepik Province.

§ Enteropathogenic *Escherichia coli* (EPEC); enterotoxigenic *Escherichia coli* (ETEC).

**Figure 1.**
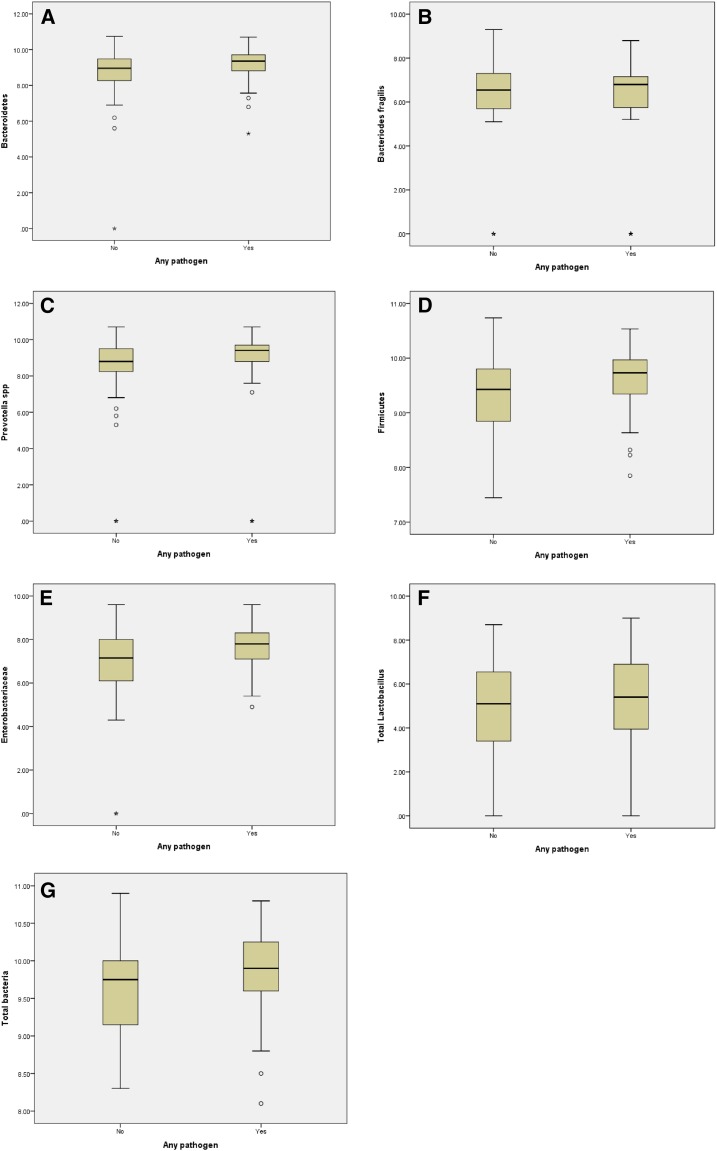
Box plots comparing numbers of key bacterial communities in carriage positive and carriage negative individuals. (**A**) Bacteriodetes vs. any pathogen; (**B**) *Bacteroides fragilis* vs. any pathogen; (**C**) *Prevotella* spp. vs. any pathogen; (**D**) Firmicutes vs. any pathogen; (**E**) Enterobacteriaceae vs. any pathogen; (**F**) Total *Lactobacillus* vs. any pathogen; (**G**) Total bacteria vs. any pathogen. This figure appears in color at www.ajtmh.org.

Our study detected unexpectedly high rates of pathogen carriage, particularly norovirus and shigella, in asymptomatic people in the highland communities in Papua New Guinea. Neither norovirus GII nor *Shigella* are typically associated with asymptomatic carriage. The Global Enteric Multicenter Study found only low asymptomatic carriage of both norovirus GII and *Shigella* spp. in healthy controls.^9^ A study conducted in 399 healthy adults in Australia found no asymptomatic carriage of noroviruses in that population.^10^ To date, there has been only one diarrheal etiology study in Papua New Guinea that included noroviruses in the testing. This study found that norovirus GII and norovirus GI were present in 6% and 3.5% of pediatric hospitalizations associated with acute watery diarrhea, respectively.^7^

The most concerning finding of this study is the evidence of a high rate of asymptomatic carriage of *Shigella* in highland communities. The Global Burden of Disease Study determined that shigellosis was one of the most important contributors to all-age DALYs in both 1990 and 2010, and within the diarrheal diseases, it was the second leading cause of all-age deaths.^11,12^ Similarly, the recent Global Enteric Multicenter Study conducted in four African and three Asian nations showed *Shigella* to be a significant cause of moderate to severe diarrhea.^9^ *Shigella* is arguably the leading cause of moderate to severe diarrhea/dysentery in adults globally; and with the recommendation by WHO for the introduction of the rotavirus vaccine in all countries,^13^ *Shigella* is likely to become the leading cause of serious diarrheal illness and death due to diarrhea in children globally. Furthermore, the clinical management of *Shigella* is increasingly challenging because of the widespread prevalence of antibiotic-resistant strains.^14^

Evidence presented here along with previous data from diarrheal etiology studies suggests that this setting is highly endemic for *Shigella*; resulting in high exposure and temporary carriage. Currently, there is no reliable way to distinguish *Shigella* from EIEC when using molecular detection directly on stool samples, as both pathogens contain the *ipah* gene. However, EIEC is rarely isolated in Papua New Guinea. The limited number of diarrheal etiology studies conducted to date in Papua New Guinea provide evidence that *Shigella* spp. are among the most common cause of diarrheal illnesses, for both children^7,15^ and the general population.^16^ Indeed, this pathogen has also been linked with large, mortality-associated outbreaks in displaced populations in this country.^17^ Because of the cross-sectional design of this study, we cannot account for dynamic fluctuations in microbiota “normal flora” and enteric pathogen carriage. Indeed, persistent shedding of *Shigella* sp. has been documented in some people after infection,^18^ so a recent outbreak of *Shigella* in highland communities > 2 weeks before the sampling missions cannot be discounted. However, the high rate of *Shigella* positivity in both highland communities, separated by considerable distance geographically (> 14 hours by road transport) and both relatively isolated from mainstream PNG society, would suggest that any such outbreaks would be unrelated. Further analysis on other remote highland communities, ideally with larger cohorts of participants and longitudinal sampling, are needed to further investigate the mechanism responsible for this high carriage rate and the possibility of an association with gut microbiota.

The reason for disparities in pathogen detection between highland and lowland communities is not known. Our analyses did not suggest that gut microbial composition was directly associated with pathogen carriage. However, our previous research revealed differences in the microbial community composition between highland and lowland people.^4^ This observation is based on quantitative PCR analysis of selected species/genera of the gut microbial community. There are limitations to this approach compared with 16S metagenomic sequence analysis. Although the targeted PCR approach undertaken in our previous study is sufficiently sensitive to detect community differences, it may be unable to detect subtle difference in community composition that may be present in carriage-positive participants compared with carriage negative participants.

Water, hygiene, and sanitation data were not collected as part of this study; however, it is conceivable that this was a factor. Customs and practices differ among the different language groups in PNG; as well as access to improved water. In communities where there is poor sanitation and limited access to safe water sources, there is continual exposure to enteric pathogens. As a result, there is a much higher burden of diarrheal and other enteric illnesses due to the constant consumption of these organisms. Papua New Guinea has the highest incidence of diarrhea in the Western Pacific Region for children < 5 years old; moreover, the decline in diarrheal incidence between 1990 and 2010 in Papua New Guinea has been modest by regional and global standards.^19^ Recent reports have stated that Papua New Guinea is one of only three countries globally with rates of access to safe water sources below 50%^20^, which undoubtedly contributed to the recent cholera outbreak in the country.^21^ Further research is needed to determine the factors leading to the high rates of pathogen carriage observed in the highland populations included in this study.
